# Defective lipid metabolism associated with mutation in *klf-2* and *klf-3*: important roles of essential dietary salts in fat storage

**DOI:** 10.1186/s12986-017-0172-8

**Published:** 2017-02-28

**Authors:** Jun Ling, Christopher Brey, Megan Schilling, Farah Lateef, Zenaida P. Lopez-Dee, Kristopher Fernandes, Kavita Thiruchelvam, Yi Wang, Kshitij Chandel, Kai Rau, Ranjit Parhar, Futwan Al-Mohanna, Randy Gaugler, Sarwar Hashmi

**Affiliations:** 1 Department of Basic Sciences, Geisinger Commonwealth School of Medicine, 525 Pine Street, Scranton, PA 18509 USA; 2 Science Department, Marywood University, Scranton, PA 18509 USA; 3 Molecular, Cellular and Integrative Biosciences Huck Institute of the Life Sciences, The Pennsylvania State University, University Park, PA 16802 USA; 4 Department of Cell Biology-Cardiovascular unit, KFSH&RC, Riyadh, Saudi Arabia; 5 Laboratory of Developmental Biology, Center for Vector Biology, Rutgers University, 180, Jones Avenue, New Brunswick, NJ 08901 USA; 6 Rutgers Center for Lipid Research, New Jersey Institute for Food, Nutrition, & Health, Rutgers University, New Brunswick, NJ 08901 USA

**Keywords:** *Caenorhabditis elegans (C. elegans)*, Dietary salt, Lipid metabolism, Krüppel like factor (KLF), KLF*-2*, KLF*-3*, KLF mutant

## Abstract

**Background:**

Dietary salts are important factors in metabolic disorders. They are vital components of enzymes, vitamins, hormones, and signal transduction that act synergistically to regulate lipid metabolism. Our previous studies have identified that Krüppel-like factor −3 (KLF-3) is an essential regulator of lipid metabolism. However, it is not known if KLF-2 also regulates lipid metabolism and whether KLF-2 and −3 mediate the effects of dietary salts on lipid metabolism.

**Methods:**

In this study, we used *klf* mutants [homozygous *klf-2* (ok1043) V and *klf-3* (ok1975) II mutants] to investigate the role of dietary salts in lipid metabolism. All gene expression was quantified by qRT-PCR. Localization of KLF-2 was analyzed by the expression of *klf-2*::*gfp* (in pPD95.75 vector) using a fluorescent microscope. Fat storage was measured by Oil Red O staining.

**Results:**

*Klf-2* was identified to express in the intestine during all stages of *Caenorhabditis elegans* development with peak expression at L3 stage. Mutation of *klf-2* increased fat accumulation. Under regular growth media free of Ca^2+,^ the expression of both *klf-2* and −*3* was inhibited slightly; further their expression reduced significantly in WT worms fed on 10X Ca^2+^ diet. When *klf-3* was mutated, the expression of *klf-2* increased under 10X Ca^2+^ diet; but when *klf-2* was mutated, the expression of *klf-3* was not altered under 10X Ca^2+^ diet. Overall, Mg^2+^ and K^+^ were less effective on the gene expression of *klfs*. KLF target gene *Ce*-C/EBP-2 showed elevated expression in WT and *klf-3* (ok1975) worms with changed Ca^2+^ concentrations but not in *klf-2* (ok1043) worms. However, high Ca^+2^ diet exhibited inhibitory effect on *Ce*-SREBP expression in WT worms.

**Conclusion:**

Dietary Ca^2+^ is most effective on fat storage and *klf-2* expression, wherein high Ca^2+^ diet decreased *klf-2* expression and reduced fat buildup. Mechanistic study identified *Ce*-C/EBP (C48E7.3; *lpd-2*) and Ce-SREBP (Y47D3B.7; *lpd-1*) as the target genes of *klf-2* and/or *klf-3* to mediate lipid metabolism. This study identifies a new function of *klf-2* in inhibiting fat buildup and reveals the interplay between dietary salts and *klf-2* and *klf-3* in lipid metabolism.

## Background

In humans, lipid metabolism disorder can result in fat buildup in adipose and other tissues causing obesity and diabetes. Several recent reports also suggest that fat build up in adipose tissues is one of the important factors that may lead to many types of cancers, such as cancer of colon, breast, gallbladder, ovaries, pancreas, kidney, and esophagus [1]. Obesity, diabetes and heart diseases are inseparably linked to consumption of fatty food and/or irregularities in the use of common dietary salts, including sodium, calcium, magnesium, and potassium. The epidemiological and clinical studies along with experimental studies involving animal models have identified important functions of dietary salts in biological systems [2–4]. Dietary salts function through enzymes, vitamins, hormones and signal transduction to collectively regulate lipid metabolism.

Lipid metabolism is regulated by a complex network of hormones and transcription factors. Mammalian Krüppel-like transcription factors (KLFs) are known to perform critical functions in lipid metabolism and lipogenesis in adipose and non-adipose tissues (pancreas, liver or muscle). KLFs belong to a family of Sp1-like zinc-finger proteins [5–11]. KLFs bind GC/GT-rich promoter elements through three C_2_H_2_-type zinc fingers at their C-terminal domains. The KLF proteins are key regulators of respiratory, hematological, and immune systems. Their dysregulation can lead to many serious human diseases [12–16], which are supported by molecular mechanisms that KLFs can regulate for example cell differentiation, proliferation, signal transduction, adipogenesis, and apoptosis [17–20]

Our previous studies were focused on molecular genetics and physiological functions of *Caenorhabditis elegans klfs*, *klf-1* [21] and *klf-3* [22–24]. *Ce*-KLFs share high identity with members of mammalian KLFs in terms of their C-terminal C_2_H_2_ zinc fingers, despite little homology in their N-terminal regions. Previously we have shown that *C. elegans klf-1* regulates fat metabolism, programmed cell death and phagocytosis [21]. Recently several studies conducted in our lab on KLF-3 protein also demonstrated that worm *klf-3* is an important regulator of fatty acid synthesis, lipid secretion and degradation that are critical steps in mammalian lipid metabolism [22–26]. *C. elegans klfs*-*1*, −*2*, and −*3* specifically express in the intestine and during all developmental stages of worm. The intestinal expression of KLFs is significant because intestine is the major site of lipid metabolism in *C. elegans*. The *C. elegans* feeds on bacteria *Escherichia coli* that are grown on Nutrient Growth Media (NGM) supplemented with a measurable quantity of Ca^2+^ Mg^2+^, and K^+^, and Na^+^. These dietary salts play essential roles in the development and metabolisms of *C. elegans* [27]. However, it is not known if dietary salts affect lipid metabolism through the regulation of *klfs* and if *klf-2* is also involved in lipid metabolism. Here, we utilize deletion mutation as an effective approach in *C. elegans* to address this topic.

In this study, we focused on the role of *klf-2* in the regulation of lipid metabolism. *C. elegan*s *klf-2* (ok1043) was used to determine if Ca^2+^, Mg^2+^, and K^+^ have effects on lipid metabolism, *klf-3* (ok1975) mutant was included as a comparison. *Klf-2* was identified to express in the intestine which is consistent with their spatiotemporal expression during development, implying its regulatory role in lipid metabolism. Calcium was identified to be most effective in regulating fat storage and *klf-2* expression, wherein high Ca^2+^ diet decreased *klf-2* expression and reduced fat buildup. This finding is similar to our previous study on *klf-3*, thus revealing that *klf-2* plays a similar role in lipid metabolism but with different quantitative and developmental pattern as compared to *klf-3*. Mechanistic study identified that *Ce*-C/EBP (C48E7.3; *lpd-2*) and *Ce*-SREBP (Y47D3B.7; *lpd-1*) are the target genes of *klf-2* and/or *klf-3* to mediate lipid metabolism. Overall, this study identifies a new function of *klf-2* in regulating fat buildup in response to various dietary salt conditions. The results from this study also advance our understanding of the regulation of lipid metabolism by dietary salts at gene transcriptional level via *klf*s.

## Methods

### Nematode strains and culture conditions

All *C. elegan* strains used in this study were maintained and propagated at 20 °C on Petri plates containing NGM seeded with the *E. coli* strain OP50 [28]. The wild-type (WT) strain N2 (Bristol) was used to create transgenic strains. The homozygous *klf-2* (ok1043) V and *klf-3* (ok1975) II mutants were generated by OMRF knockout group and provided by *C. elegans* Genetics Center (Minneapolis, MN, USA), which is funded by the NIH National Center for Research Resources. The genomic deletion or mutation in mutant strains used in this study was confirmed by nested PCR and DNA sequencing. We note that *klf-2* (ok1043) allele listed in Wormbase (http://legacy.wormbase.org/) with ~1.5 kb bp deletion actually harbors a 2.1 kb deletion.

### Analysis of *klf-2* (ok1043) allele

To characterize *Ce*-*klf2* (ok1043) mutant, the mutant strain was backcrossed 3 times using wild type N2 (Bristol) strain males according to a standard protocol [28] and maintained as homozygous worms. Using single worm PCR, ~10 individual homozygous mutant alleles were individually sequenced to confirm the deletion site. Mutant was rescued by injecting the full coding sequence of the wild type copy of the *klf-2* gene into the young *klf-2* (ok1043) hermaphrodite’s gonads. Individual homozygous mutant hermaphrodites were grown on plates at 22 °C and their self-progenies were used in subsequent experiments. To measure fertility, 25 individual L1/L2 larvae were separately placed onto NGM plates, their growth and development was observed at room temperature (22 °C). When these worms began to lay egg, the number of embryos produced by each of these worms was counted. Individual worms were transferred to fresh NGM plates every 24 h followed by counting the eggs and larvae for five consecutive days.

### Expression of *klf-2*::*gfp* in *C. elegans*

**Fig. 1:**
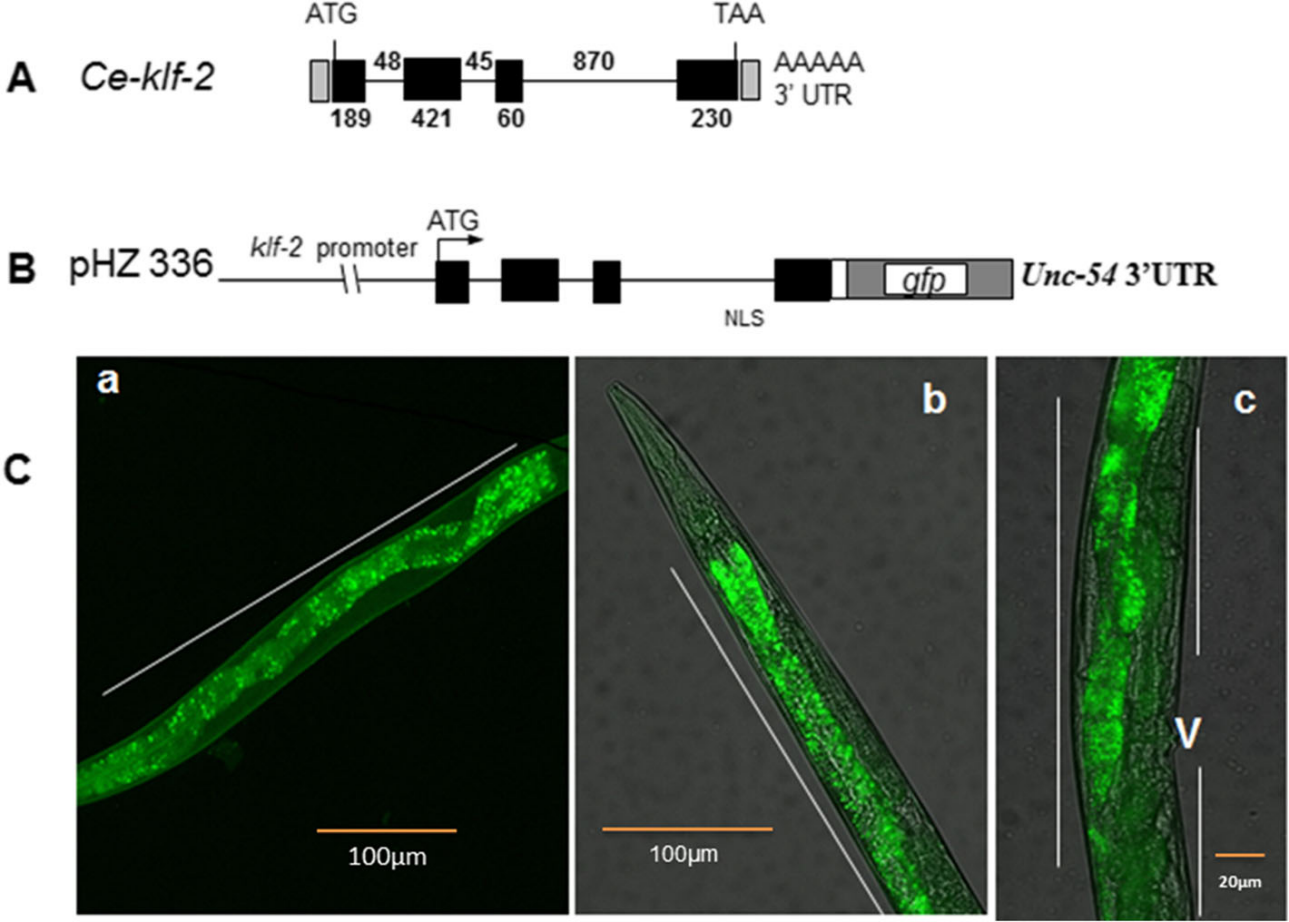
Analysis of *klf-2* expression with GFP reporter during *C. elegans* development. Lines of transgenic *C. elega*ns carrying the *klf-2::gfp* reporter gene was created as described in the “Methods”. **a** the structure of *klf-2* gene; **b** the *klf-2::g*fp reporter gene construct; **c** the fluorescence microscopic images *of klf-2* expression: *a*) The *gfp* expression was observed along the length of early larval (L1-L2) intestine (solid line); *b*) GFP is present along the length of the intestine of young adult hermaphrodites (solid line); and *c*) GFP is present along the length of intestine (one long solid line) in egg-laying hermaphrodites. GFP expression is not present in gonads (short solid lines) and vulva (v). Transgenic *C. elegans worms* were observed and photographed using Axioskop 2 plus fluorescent microscope (Zeiss, Germany) with appropriate filter sets for GFP (magnification: 200X)

To study the expression and localization of *klf-2* in developing worm, we made a translational fusion construct that contained the 5’ flanking genomic sequences (~2 kb) from *klf-2* ATG and the full coding sequences covering all its 4 exons (Fig. 1a). The promoter region along with the coding sequences was PCR amplified, and cloned into *C. elegans* expression vector pPD95_75 (a gift from Andrew Fire, Addgene plasmid #1494) containing green fluorescent protein (*gfp*) as reporter gene. The resulting *klf-2*::*gfp* construct was designated as pHZ336 (Fig. 1b) and sequenced. The plasmid DNA for injection were prepared using the Concert™ rapid plasmid miniprep system (Gibco, BRL, Rockville, MD), and then injected into the gonadal syntium of *C. elegans* young adult hermaphrodites [29] at a concentration of 50 ng/μl. A plasmid DNA (pRF4) containing the dominant selectable marker gene rol-6 (su1006), which encodes a mutant collagen was also co-injected (50 ng/μl) with the reporter construct. When worms express the rol-6 gene, they continuously rolls over, thus provide a visible phenotype for the selection of transgenic worms. The F3 generation worms showing a roller phenotype were collected to observe for *gfp* expression under fluorescent microscope. At least three independent lines (approximately 200 worms for each line) were examined for each construct.

### Stage-specific profile of the *klf-2* mRNA using qRT- PCR

We used real-time quantitative PCR (qRT-PCR) to determine the stage-specific expression profiles of *klf-2* in worm during development. Synchronous population of all four larval stages and adult population were generated as described by Sulston and Hodgkin [30]. In brief, embryos obtained by treatment of gravid hermaphrodites with sodium hypochlorite were washed with water and transferred to a glass cavity block containing water to hatch into first-stage larvae (L1) overnight [31]. The arrested L1 larvae were transferred onto NGM agarose plates seeded with OP50 bacteria, which allowed the L1 larvae to develop into L2, L3, L4 and adult over 40 h. Total RNA was prepared from embryos and from larvae L1, L2, L3, L4 and adult stages using Pure Link RNA Mini Kit (Ambion, Grand Island, NY) according to the manufacturer’s protocol. Messenger RNA was converted to cDNA using AMV First Strand cDNA Synthesis Kit (NE BioLabs Inc., Ipswich, MA). The Power SYBR® Green kit (Applied Biosystems, Life Technologies, Carlsbad, CA) was used for qRT-PCR reactions run on ABI 7500 Fast system. Each sample was repeated 3 times; the expression of *ama-1* [32, 33] was used as the internal control to normalize the expression of target genes. The qRT-PCR data were analyzed by ABI7500 Fast software. The linear fold change (RQ = 2^-(ddCt) was calculated to compare the difference between each group. The primers are CTTGGCGATTTGCACGATCC (forward) and ATCCGTTTCATGCCGCTTCA (reverse) for *Ce-klf-2*, and 5’-CGGATGGAGGAGCATCGCCG-3’ (forward) and 5’-CAGCGGCTGGGGAAGTTGGC-3’ (reverse) for *ama-1* [32].

### Effect of dietary salts on genes expression

To test the effects of dietary salts on worms, three sets of NGM agarose plates were set-up in two replicates; one set of NGM-agarose plates, containing a normal quantity of Ca^2+^ (1 M CaCl_2_), Mg^2+^ (1 M Mg_2_SO_4_) and K^+^ (1 M K_2_HPO_4_) that is used in NGM media; second set of NGM plates with media free from Ca^2+^, Mg^2+^ or K^+^; and the third set of NGM plates added with 10X Ca^2+^, Mg^2+^ or K^+^ salt. Then, ~100 young larvae (L1-L2s) of *klf-2* (ok1043) or *klf-3* (ok1975) or WT worms were seeded on each plate. After 5–6 days when the larvae became adults and produced F1 progenies, all worms (~90% L1-L2s) were collected in Eppendorf tube by washing and decanting using PBS (Phosphate Buffer Saline). The RNA extraction, cDNA synthesis, and qRT-PCR reaction and data analysis were carried out as same as above. The primers for *klf-3 are*: ATG GAA CAA AGT GCA CCT CCA (forward); TTT CTG CTC GAG TCC CTT TCA (reverse). The primers for *Ce*-SREBP (Y47D3B.7; *lpd-1*) and *Ce*-C/EBP (C48E7.3; *lpd-2*) are CEBP-2: [(forward)/TGAGTGGAAATCGGAAGCGAA; (reverse)/TCGGGTTCTGTTCACAGCTTC and SREBP-1: [(forward)/AGCATCAGGTGTGGTGTCTG; (reverse)/CGTCGAGCAGCGAGTTCATA].

### Oil Red O staining for the measurement of fat buildup

We used Oil Red O staining procedures to examine fat buildup in worms. Larval stages (~1000 L1-L2 larvae) wild type, *klf-2* (ok1043) and *klf-3* (ok1975) worms were separately collected by washing with PBS from NGM plates to a 2-ml Eppendorf tube as described previously [26]. After collection, worms were washed twice and re-suspended with 200 μl of PBS buffer. Worms were fixed in 1% formaldehyde in PBS for 1 h at room temperature, kept overnight at −80 °C, and thawed under a stream of running tap water, followed by addition of 1 ml of distilled water. Samples were mixed and collected by centrifugation. One milliliter of propylene glycol was added to the tube containing the sample and incubated at room temperature (∼22 °C) for 20–30 min on a gentle shaker and collected by centrifugation at ∼ 5000 rpm. Then 1 ml of pre-warmed (60 °C water bath) Oil Red O stain (STORO100; American Master Tech Scientific, Lodi, CA) was added to the sample and incubated overnight at 4 °C. After incubation, samples were brought to room temperature with gentle shaking and transferred to a glass well/wash plate (Pyrex plate, cat no. 71563; Electron Microscopy Sciences, Hatfield, PA). The stained worms were transferred to a tiny drop of propylene glycol on a 2% agarose pad on a glass slides, covered with coverslip and observed under a light microscope equipped with DIC optics.

### Effect of dietary salts on fat buildup in *klf-2* (ok1043) and *klf-3* (ok1975) worms

To test the effects of various concentrations of Ca^2+^, Mg^2+^ or K^+^ on fat buildup, worms were treated and cultured as same as described in “Effect of dietary salts on gene expression” section, followed by the Oil Red O staining as described above. Three slides, each containing ~50-60 worms were prepared for each sample. At least two independent experiments were performed. For each treatment ~150 worms were observed using 20X objectives and imaged at 200X magnification. Almost 98% of all worms showed similar staining pattern. Images representative of ~ 150 worms are shown. For quantitative analysis, we measured the integrated density of Oil Red O staining in 10 worms from each treatment using Photoshop CS3 (extended) software.

### Statistical analysis

Data were subjected to statistical analysis using analysis of variance (ANOVA). Fisher’s least significant difference (LSD) test was applied to separate means at a 95% confidence level. Data is presented as mean ± standard deviation.

## Results

### K*lf-2* is predominantly expressed in intestinal cells

We injected a *klf-2*::*gfp* construct in WT young *C. elegans* hermaphrodite and established several transgenic lines to examine the expression profile of *klf-2* during worm development. As indicated by *gfp,* the expression of *klf-2* was not noticeable in embryos (data not shown) but prominent in all four larval and adult stages. During larval development, the *gfp* expression was continuously observed along the length of the intestine during the developing larva and adult stages (Fig. 1c), suggesting that *klf-2* is mainly located in intestine. The *C. elegans* intestine is known to perform many important functions including digestion, fat storage and distribution, thus providing a basis to investigate *klf-2* function and its interaction with dietary salts in lipid metabolism.

### *Klf-2* is differentially expressed during all developmental stages of the worm

**Fig. 2:**
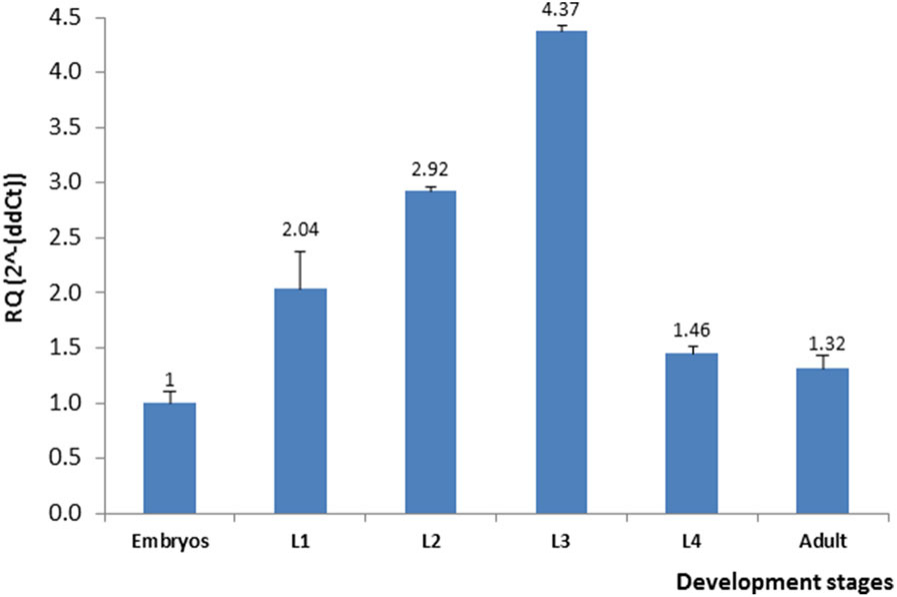
Temporal pattern of *klf-2* expression as determined by qRT-PCR. The levels of *klf-2* mRNA transcript in each developmental stage were measured by qRT-PCR as described in the “Methods”. The expression of *klf-2* was normalized against the internal control *ama-1* expression, and the linear fold-change (RQ = 2^-(ddCt) was presented with the mean ± standard error. Each experimental point was repeated three times

We examined the mRNA transcripts of *klf-2* in embryos, larvae and adult stages of WT worm during development using qRT-PCR. The gene *ama-1* was used as an internal control to enable a relative and accurate quantification of *klf-2* expression. The data obtained through qRT-PCR provided a measurement for the relative abundance of *klf-2* transcripts in various developmental stages for comparative analysis during *C. elegans* development. As shown in Fig. 2, a convincing and reproducible gene expression data was obtained for various developmental stages of the worm. *klf-2* was found to express in all developmental stages, however, its expression was gradually increased from embryo to L3, followed by a decrease in L4 and stabilized in adult stage. This expression pattern during development stages is somewhat different from *klf-3* expression [22], suggesting that *klf-2* and *klf-3* may have different functions or exhibit partially redundant functions but at different stages of development.

### Mutation in *klf-2* results in excessive fat builds up and reduced reproduction

Through genetic analysis, we identified that the *C. elegans klf-2* (ok1043) mutant actually contains a 2.1 kb deletion in the *klf-2* gene that includes 1186 bp upstream sequence from ATG; this upstream region may include a portion of *klf-2* putative promoter. The rest of 2.1 kb sequence includes a major portion of *klf-2* coding sequences that contains the sequence from exon 1 to exon 3. The confirmation of this *klf-2* mutant provided a molecular foundation for us to analyze its functions.

**Fig. 3:**
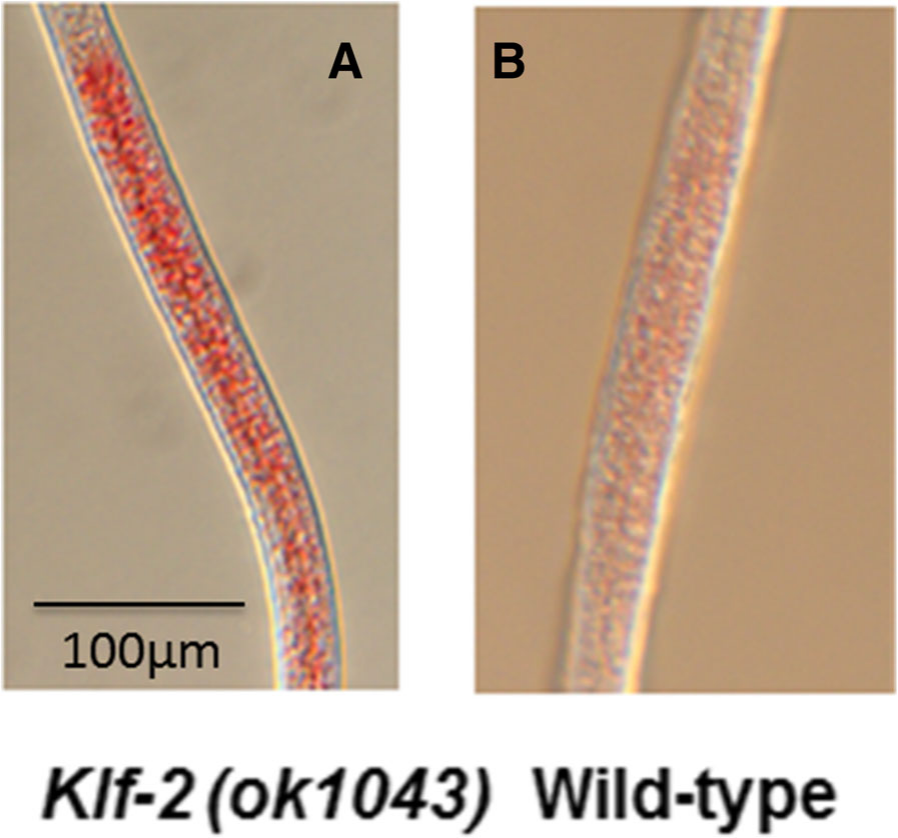
Mutation of *klf-2* results in excess fat builds up in the intestine of *klf-2* (ok1043) worm. Oil-Red-O staining was used to accurately measure fat buildup in worms carrying deletion in *klf-2* gene. **a** High fat buildup in *klf-2* (ok1043) mutant; **b** Low fat buildup in wild type worm. Worms were observed under Olympus U-Tr0.63Xc optics attached to Axioplan Zeiss microscope and images were taken with a digital camera Prog Res CF scan (magnification: 200x)

We determined if KLF-2 has activity on lipid metabolism, we used Oil Red O staining to qualitatively measure fat buildup in wild-type and in *klf-2* (ok1043) worm. The intense red staining indicated increased fat buildup in the intestine of *klf-2* (ok1043) worm. The quantitation of fat in100 each of WT and *klf-2* (ok1043) worms identified 44% increase in fat buildup in *klf-2* (ok1043) (Fig. 3 a) over WT worms (Fig. 3b), suggesting that the loss of *klf-2* activity increased fat build-up, i.e., the normal function of *klf-2* is to inhibit fat accumulation.

For biological characterization of *klf-2* mutant, we focused on its reproduction by counting the progenies produced by *klf-2* (ok1043) worms (three replicates, each containing 30 worms) from the beginning to the end of egg laying. We found that averagely *klf-2* (ok1043) worm produced 110 progenies as compared to the wild-type that produced 225 progenies during their reproductive period. Meanwhile, there were no other phenotypic changes or increased cell death in *klf-2* mutant. This finding is additionally significant as it is related with the situation in humans, where obesity is also linked to reduced reproduction [34].

### High calcium diet reduced the expression of *klf-2* and *klf-3*

**Fig. 4:**
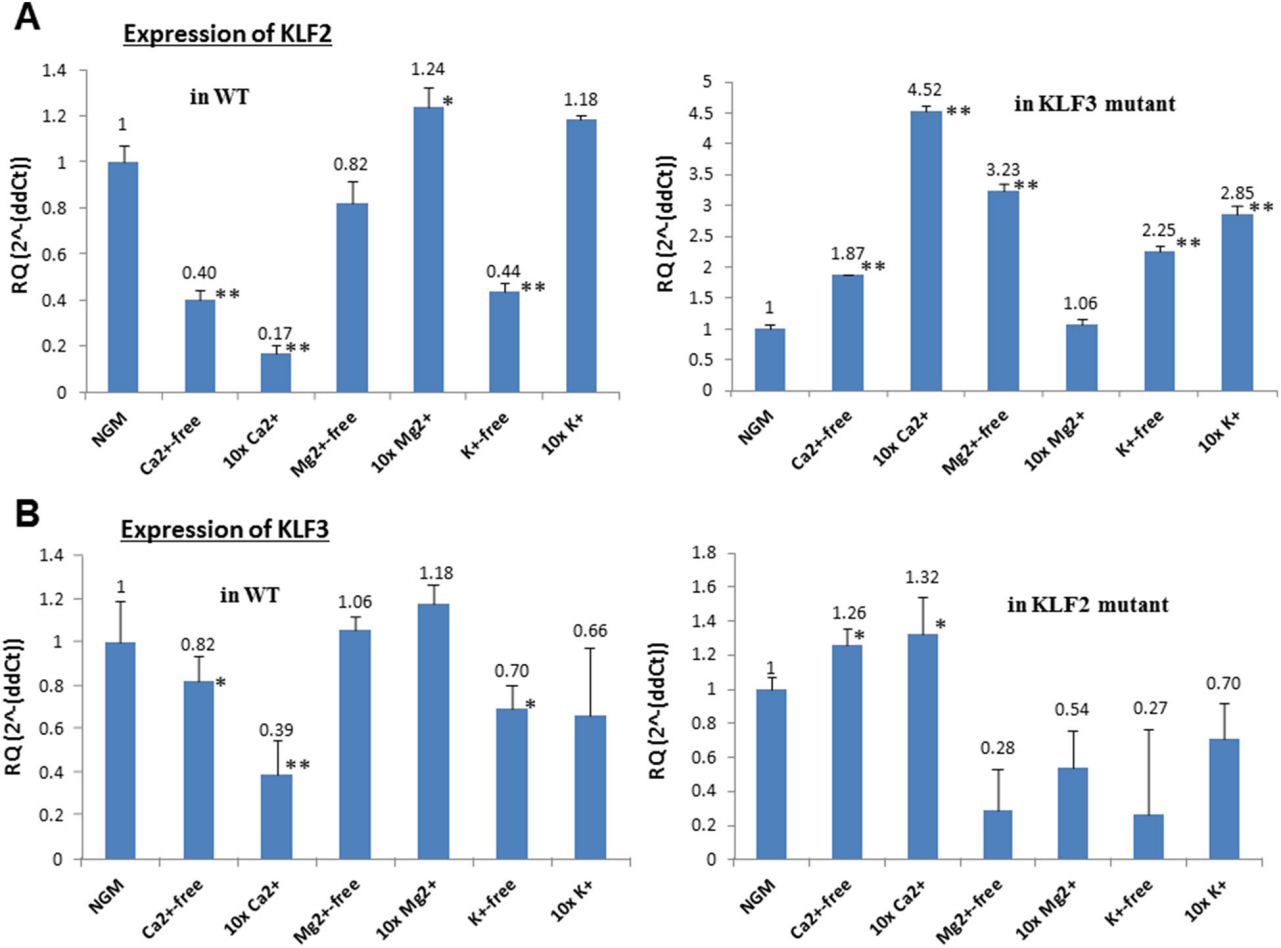
The effects of dietary salts on *klf-2* and −*3* expression in wild type and mutant *C. elegans*. Equal amounts of *C. elegans* were harvested after treatments. The mRNA purification, cDNA synthesis, qRT-PCR, and the data analysis were performed as described in the “Methods”. The mRNA levels of *klf-2* and −*3* were normalized against *ama-1* as the internal control. The linear fold-change was calculated to compare the difference between the treatments and the control (worms cultured on the regular media-NGM). **a** *klf-2* expression in WT (left panel) and *klf-3* mutant (ok1975) (right panel); **b** *klf-3* expression in WT (left panel) and *klf-2* mutant (ok1043) (right panel). Each treatment was repeated three times, and the means with standard errors were presented in all panels. * *p* < 0.05 and ** *p* < 0.01, indicating significant difference as compared to the control (NGM)

We used qRT-PCR to quantify the expression levels of *klf-2* in WT and *klf-3* (ok1975) worm fed on NGM agar media containing various concentrations of Ca^2+^, Mg ^+2,^ or K^+^. We found that the expression of *klf-2* was reduced by 60% and 83% at Ca^2+^-free and 10X Ca^2+^ media respectively (Fig. 4 a). Meanwhile, Mg^2+^ concentrations didn’t affect *klf-2* expression statistically, and the media free of K^+^ reduced *klf-2* expression by 56%. When *klf-3* is mutated in the *klf-*3 (ok1975) worm, it was found that mRNA levels of *klf-2* were increased by 1.87-fold, 4.52-fold, 3.23-fold, 2.25-fold, 2.85-fold under Ca^2+^-free, 10X Ca^2+^, Mg^2+^-free, K^+^-free, and 10X K^+^ (Fig. 4a) diets respectively, suggesting that dietary salts have broad effects on *klf-2* gene expression and *klf-2* and −*3* have certain interaction at gene transcriptional level.

The expression levels of *klf-3* were also measured in WT and *klf-2* (ok1043) mutant to test a reciprocal relationship between *klf-2*. We noted a similar pattern of *klf-3* expression with *klf-2* in response to Ca^2+^; *klf-3* expression decreased by the changes of Ca^2+^ concentration in the WT but slightly increased in *klf-2* (ok1043) worm (Fig.4b). However, *klf-3* was less sensitive to Ca^2+^ concentration change than *klf-2*, suggesting the differential gene expression characteristic between these two members of *klf* genes in *C. elegans*. Mg^2+^ and K^+^ almost had no effects on *klf-3* expression in the WT, but clearly inhibited *klf-3* expression in *klf-2* (ok1043) worm. These effects were opposite to the effects of Mg^2+^ and K^+^ on the *klf-2* expression in *klf-3* (ok1075) worms, further suggesting the complexity of the interaction between *klf-2* and *klf-3* that was supported by their different responses to Ca^2+^, Mg ^2+^, or K^+^ salts.

### CEBP and SREBP are differentially regulated in worms in response to the change of calcium diet

**Fig. 5:**
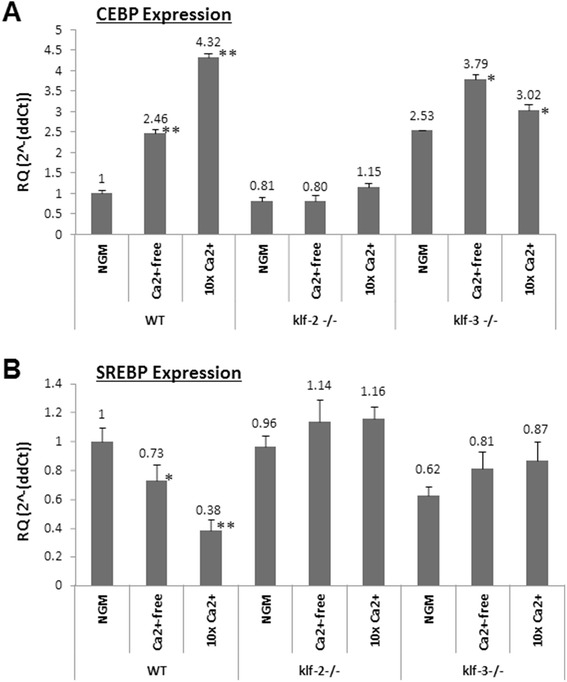
Expression of CEBP (*Ce*-lpd-2) and SREBP (*Ce*-lpd-1) in WT and *klf* mutant worms. The qRT-PCR experiments used to measure the mRNA levels of CEBP and SREBP were performed as described in the “Methods” with *ama-1* as the internal control. **a** the expression of CEBP in WT and *klf-2* and −*3* mutant worms; **b** the expression of SREBP in WT and *klf-2* and −*3* worms. The linear fold-changes were shown by the means with standard errors. Each experiment was repeated three times. * *p* < 0.05 and ** *p* < 0.01, indicating significant difference as compared to the control (NGM) in each group

High calcium diet reduced both *klf-2* and *klf-3* expression in WT worms, we ask if this reduction leads to changes in their target gene expression associated with lipid metabolism. CEBP (CCAAT/enhancer-binding proteins) and SREBP (Sterol regulatory element binding proteins) are essential regulators of lipid metabolism, and they are also KLF target genes. We then tested the expression of *C. elegans* homologs of these two genes, *Ce*-SREBP (Y47D3B.7; *lpd-1*) and *Ce*-C/EBP (C48E7.3; *lpd-2*) in WT, *klf-2* (ok1043) and *klf-3* (ok1975) worms. We found that the expression of CEBP (*Ce-lpd-2*) was up-regulated in WT worms by various concentrations of Ca^2+^ but not in *klf-2* (ok1043); it was also slightly up-regulated in *klf-3* (ok1975) worms fed on the same diet (Fig. 5a), suggesting the up-regulation of CEBP (*Ce-lpd-2*) expression in lipid formation may be more dependent on *klf-2* than *klf-3*. On the other hand, calcium diet change was found to down-regulate SREBP (*Ce*-*lpd-1*) expression in WT worms with 62% inhibition at 10X Ca^2+^ condition. Meanwhile, no remarked effects on SREBP were observed in klf-2 or klf-3 mutant worms, suggesting that the regulation of SREBP by calcium may also depend on klf-2 and/or klf-3. This result indicated that SREBP is also involved in lipid formation but in an opposite way to CEBP. Thus, the synergistic effect between CEBP and SREBP might be a factor to determine the direction and degree of lipid metabolism.

### Dietary salts show broad effect on fat buildup in *klf-2* (ok1043) and *klf-3* (ok1975) worms

**Fig. 6:**
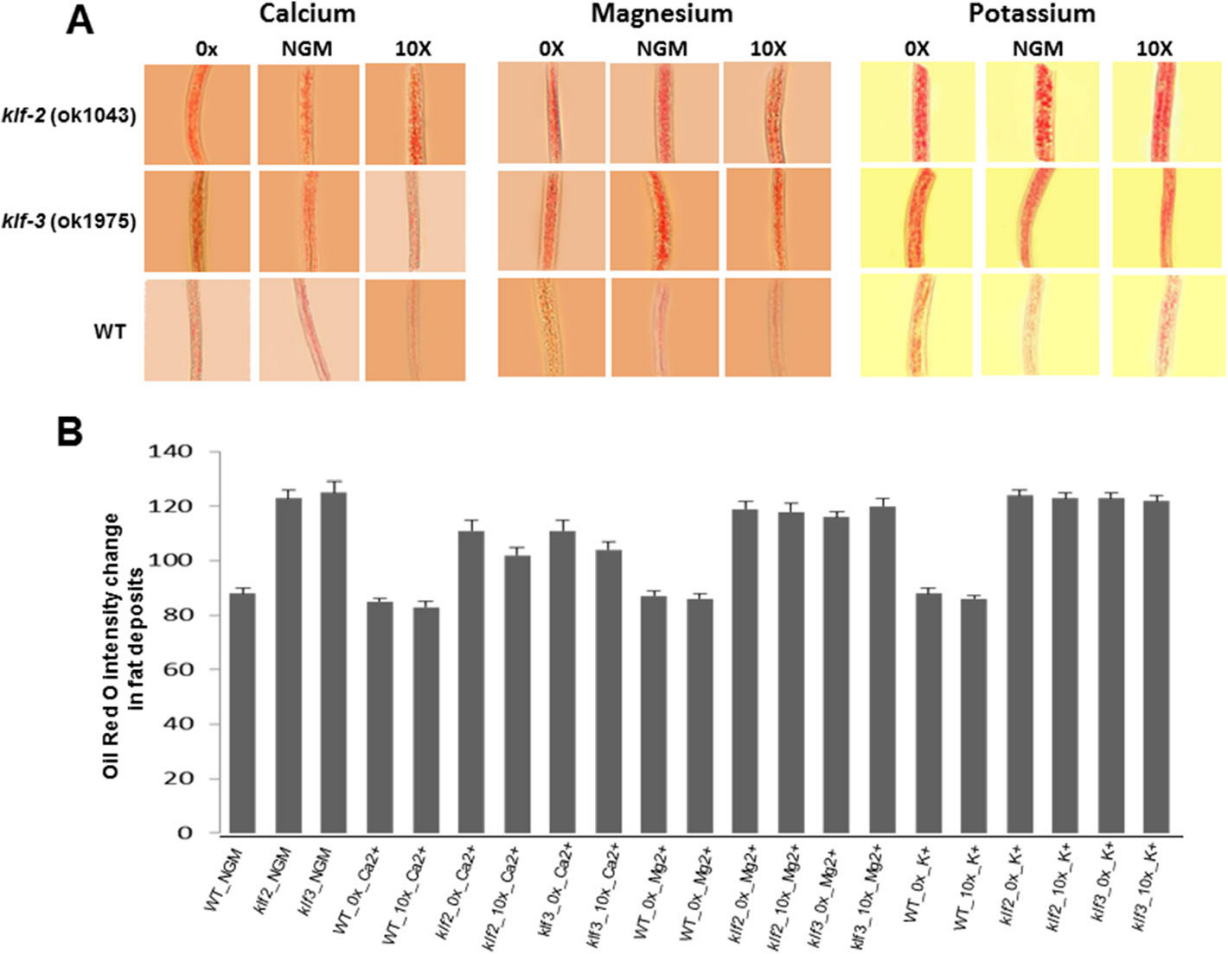
The effects of deficiency and excess of dietary salts on fat deposition in WT and *klf* mutant worms. **a** Worms were fed on normal NGM media or media with various salt concentrations (Ca^2+^, Mg^2+^, or K^+^-free, and 10x of each salt). Oil red O staining was used to measure fat mass in WT and mutant strains. Worms were observed under Olympus U-Tr0.63Xc optics attached to Axioplan Zeiss microscope and photographed using a digital camera Prog Res CF scan (magnification: 200X). Representative images were shown. **b** The quantitation of fat mass based on Oil-Red staining intensity. Multiple representative images from each treatment were chosen for image quantitation as described in the “Methods”, and the means with standard errors were presented in the chart. *p* < 0.05 when comparing the fat mass in mutants over WT worms grown on NGM, 0x (Ca^2+^, Mg^2+^, or K^+^-free media), and 10x salt media under each salt condition. Since the WT and *klf* mutant worms on NGM are identical among three salt conditions, there are 21 bars in panel B instead of 27 corresponding to the number of images in panel A

Dietary salts, calcium, magnesium and potassium regulate cellular and physiological activities and play a key regulatory role in lipid metabolism. In mammals, high-calcium diets reduce adipocyte fat buildup and weight gain during overconsumption of an energy-rich diet and increase lipolysis thereby markedly favoring weight loss. Based on the results of dietary salts on *klf-2* and −*3* expressions, we examined their effects on fat deposits in WT, *klf-2* (ok1043) and *klf-3* (ok1975) worms. It was found that 10X Ca^2+^ reduced fat buildup by 17% in *klf2* (ok1043) and by 16% in *klf-3* (ok1975) worms (Fig. 6) as compared to those worms fed on normal diet. Interestingly calcium deficient diets also reduced the fat deposits by 8% equally in *klf-2* (ok1043) and in *klf-3* (ok1975) worms; the underlying mechanisms are under study in another ongoing project. Because *klf* mutant worms have higher basal level of fat buildup, their response to Ca^*2+*^ change can be easier to observe. In contrast, wild-type worms have less basal level of fat buildup, the reduction in response to Ca^*2+*^ changes was not significant. Different from the stronger effect of Ca^2+^ on the reduction of fat buildup, feeding these worms on 10X Mg^2+^diet showed a little reduction in their fat deposits in the intestine; the fat buildup in both *klf-3* (ok1975) and *klf-2* (ok1043) worms were reduced by 5% equally as compared to the controls on normal NGM media. The Oil Red O staining pattern was almost equally intense in all worms fed on NGM, K^+^-free media, and 10X K^+^ diets; there was no significant difference in fat buildup in WT and *klf* mutant worms under all K^+^ conditions, suggesting that K^+^ might not be a significant dietary salt in regulating lipid synthesis in *C. elegans*.

## Discussion

We have identified important roles of dietary salts Ca^2+^, Mg^2+^, and K^+^ on *klf* expression and lipid metabolism. Mutation in *klf-2* (ok1043) (this study) or *klf-3* (ok1975) [22] results in fat build-up in the intestine, implying that loss of function of either *klf-2* or *klf-3* interrupts the normal process of lipid metabolism. The critical role of *C. elegans klfs* in lipid metabolism is also consistent with their expression in intestine. *Ce- klf-3* (ok1975) worm produce sterile and semi-sterile progenies and plays an important role in worm’s reproduction [22]. In this study, we identify that *klf-2* (ok1043) worms produce 50% less progenies than wildtype. However, unlike *klf-3* (ok1975) worms the *klf-2* (ok1043) worms are not infertile to produce sterile or semi-sterile progenies.

**Fig. 7:**
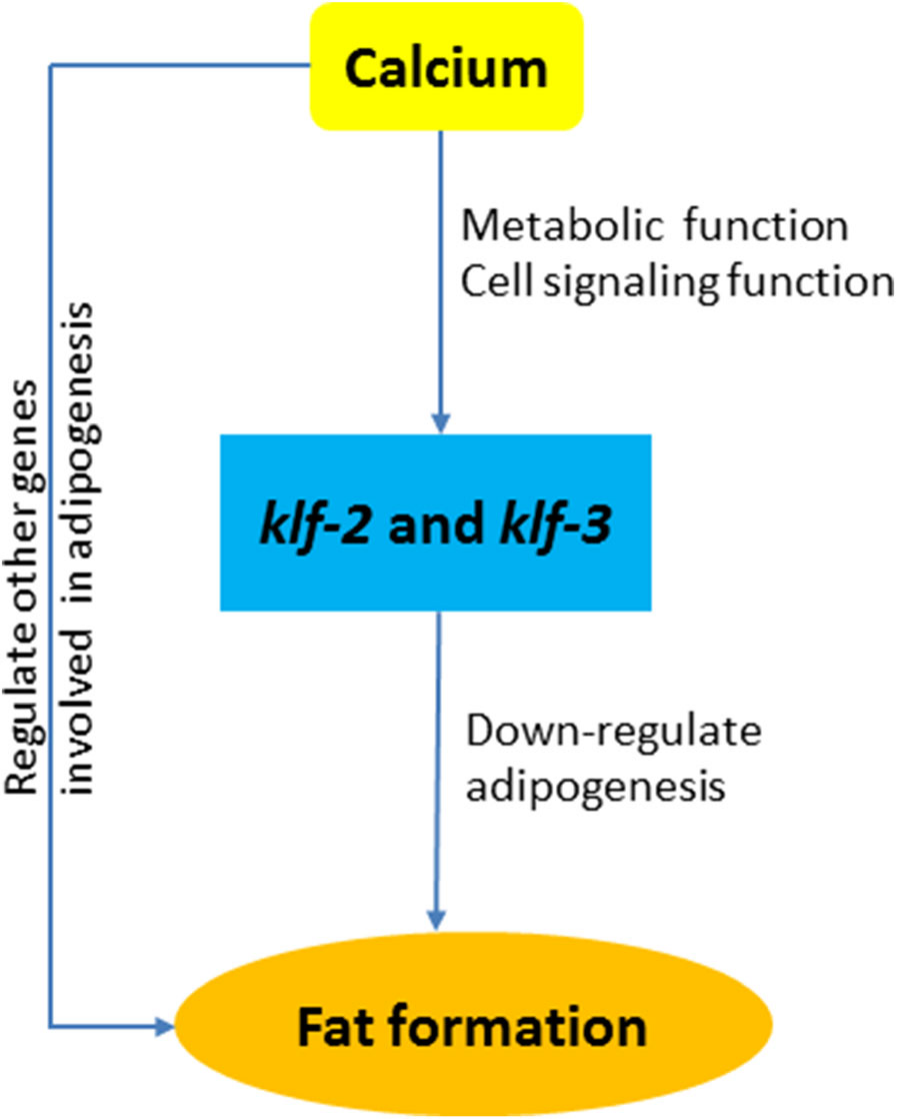
Working model of the regulation of lipid metabolism by calcium and KLFs. This model is proposed based on the experimental data and systematic analysis in this study. It aims to illustrate the relationship between calcium and KLFs and their relative contribution to fat buildup in *C. elegans*

*C. elegans* intestinal cells act as site for lipid metabolism, storage, breakdown and transport [35, 36], a complex process regulated by signal transduction and transcription factors. In this study, by taking advantage of *klf* mutants, we were able to elucidate differential effects of dietary salts on *klf-2* and −*3* expression and fat buildup. Although Mg^2+^ and K^+^ also play important roles in lipid metabolism in humans, such as Mg^2+^ required for insulin signaling and energy production [37, 38] and its deficiency associated with increased triglyceride, VLDL, LDL, triglyceride-rich lipoproteins and reduced HDL [39–41], overall these two salts do not generate remarkable effects on *klf* expression and fat buildup in worms. This conclusion is further supported by additional experiments with both Mg^2+^ and K^+^ removed or both salts increased by 10-fold, wherein there are no difference between these two settings (data not shown). In contrast, Ca^2+^ is more effective on *klf* expression and fat buildup. However, based on the reverse relationship between *klf-2* or −*3* expression and fat buildup, the effect of Ca^2+^ on *klf-2* and *klf-3* expression is not much correlated with its effect on fat accumulation, suggesting that Ca^2+^ might function partially through *klf* pathway to regulate lipid metabolism. Meanwhile, Ca^2+^ might also function through its second messenger role in many cell signaling pathways to regulate fat buildup. Thus, our overall working model for the interplay between Ca^2+^ and *klfs* on fat metabolism is proposed as depicted in Fig. 7. On the other hand, mitochondria and endoplasmic reticulum (ER) are known to be the reservoir of Ca^2+^ in eukaryotic cells to regulate intracellular Ca^2+^ concentration via Ca^2+^ itself as a second messenger [42]. Our previous study has also reported a reduced mitochondrial proliferation in *klf-2* (ok1975) mutant [26]. Increased calcium uptake is reported to suppress adipocyte intracellular Ca^2+^ and thereby synergistically regulate lipogenesis and lipolysis [43, 44]. Increased dietary calcium in human raises the serum level of calcium and seems to have a reverse relationship with intracellular calcium in a variety of cell types [45]. Thus, in our study the actual intracellular concentrations of Ca^2+^ under increased or decreased Ca^2+^ diets may be the true factor to determine the net effect of Ca^2+^ on fat accumulation.

It was intriguing to find that the expression of both *klf-2* and −*3* was repressed by the changes of Ca^2+^ concentrations in WT but promoted in *klf-3* (ok1975) and *klf-2* (ok1043) respectively. Furthermore, the down- and up-regulation of *klf-2* are quantitatively more remarkable than *klf-3*, among which 10X Ca^2+^ repressed the *klf-2* expression by ~5-fold in the WT worm but promoted its expression by 4.5-fold in the *klf-3* (ok1975) worms. This opposite effect of Ca^2+^ on the expression of *klf-2* and −*3* in WT and mutant worms suggests that *klf-2* and −*3* share similar functions in lipid metabolism and the *klf-2* expression is sensitive to Ca^2+^. This result also implies that there is a potential interaction between *klf-2* and *klf-3* at transcriptional level, which will be investigated in our future study. Theoretically, if increased Ca^2+^ (i.e.10-fold) down-regulates *klf-2* and −*3* expression, decreased Ca^2+^ (i.e. Ca^2+^-free media) should up-regulate *klf-2* and −*3* expression. However, our result is opposite, wherein both increased and decreased Ca^2+^ generate the same direction of effect, suggesting that calcium homeostasis is more important than actual concentration of intracellular Ca^2+^. When the optimal Ca^2+^ balance is broken, perturbation of Ca^2+^ concentration in either way may generate the same cellular effect.

Genetic regulation of lipogenesis is a well-studied area in mammalian systems. Transcription factors PPARγ (Peroxisome proliferator-activated receptors), C/EBPα, and the basic-helix-loop-helix protein ADD1/SREBP regulate adipogenesis [46], and their expression is inhibited by KLF2 [46] in mammal. The homolog of mammalian SREBP, *Ce-*SREBP (Y47D3B.7; *Ce-lpd-1*) is exclusively expressed in the intestines while *C. elegans* homolog of mammalian CEBP, *Ce-*C/EBP (C48E7.3; *Ce-lpd-2*) is weakly yet extensively expressed in the nervous system (http://legacy.wormbase.org/). Disruption of either C/EBP or SREBP by RNAi results in pale, skinny, lipid-depleted, and developmentally-arrested worms [47], suggesting that SREBP or C/EBP may be also essential for lipid metabolism in worms. Furthermore, SREBP and C/EBP regulate the same lipogenic enzymes in both worms and mammals [47]. Thus, our finding in this study that Ca^2+^ regulates the expression of *Ce*-CEBP and *Ce*-SREBP differentially in WT and *klf-2* and −*3* mutants is significant to link the potential crosstalk between calcium and KLF signaling. As calcium signaling is also known to be widely involved in lipogenesis and lipolysis [2, 48–50] and regulates a number of transcription factors [51–53], this study sheds new light on broad roles of KLFs in the nutritional regulation of obesity and diabetes in humans.

## Conclusions

By utilizing *C. elegans* as a model system with the advantage in genetic manipulation, we have identified a new function of KLF-2 in inhibiting lipid formation in study. The integration of dietary salts into the interaction with KLFs on lipid metabolism is a novel aspect of this study. Among the three salts examined, Ca^2+^ is most effective in regulating *klf-2* and −*3* expression and fat buildup. Changes of Ca^2+^ concentration down-regulate the expression of *klf-2* and −*3* in WT worms and up-regulate their expression in *klf* mutants. Meanwhile, CEBP and SREBP as key transcription factors for lipogenesis and the target genes of KLFs are also identified to be responsive to Ca^2+^ changes, hence defining a new topic to study the functional interaction between calcium signaling and KLFs in lipid metabolism. On the medical relevance, further understanding of the molecular mechanisms underlying the regulation of lipid metabolism by KLFs and dietary salts will promote the research of obesity related diabetes, cancers, and cardiovascular diseases.

## Abbreviations

CaSR: Calcium sensor
C/EBPα: CCAAT/enhancer-binding proteins
*C. elegans*: *Caenorhabditis elegans*
KLF: Krüppel-like factors
NGM: Nutrient growth media
PPARγ: Peroxisome proliferator-activated receptors
ADD1/SREBP: Sterol regulatory element binding proteins
PTH: Parathyroid hormone
WT: Wild type
